# Deciphering molecular mechanisms underlying chemoresistance in relapsed AML patients: towards precision medicine overcoming drug resistance

**DOI:** 10.1186/s12935-021-01746-w

**Published:** 2021-01-14

**Authors:** May Levin, Michal Stark, Yishai Ofran, Yehuda G. Assaraf

**Affiliations:** 1 The Fred Wyszkowski Cancer Research Laboratory, Dept. of Biology, Technion-Israel Institute of Technology, 3200003 Haifa, Israel; 2 Department of Hematology and Bone Marrow Transplantation, Rambam Health Care Campus, Haifa, Israel

**Keywords:** AML, Chemotherapy, Intrinsic/acquired chemoresistance, Resistance modalities, Drug metabolism, Relapse, Precision medicine

## Abstract

**Background:**

Acute myeloid leukemia (AML) remains a devastating disease with a 5-year survival rate of less than 30%. AML treatment has undergone significant changes in recent years, incorporating novel targeted therapies along with improvements in allogeneic bone marrow transplantation techniques. However, the standard of care remains cytarabine and anthracyclines, and the primary hindrance towards curative treatment is the frequent emergence of intrinsic and acquired anticancer drug resistance. In this respect, patients presenting with chemoresistant AML face dismal prognosis even with most advanced therapies. Herein, we aimed to explore the potential implementation of the characterization of chemoresistance mechanisms in individual AML patients towards efficacious personalized medicine.

**Methods:**

Towards the identification of tailored treatments for individual patients, we herein present the cases of relapsed AML patients, and compare them to patients displaying durable remissions following the same chemotherapeutic induction treatment. We quantified the expression levels of specific genes mediating drug transport and metabolism, nucleotide biosynthesis, and apoptosis, in order to decipher the molecular mechanisms underlying intrinsic and/or acquired chemoresistance modalities in relapsed patients. This was achieved by real-time PCR using patient cDNA, and could be readily implemented in the clinical setting.

**Results:**

This analysis revealed pre-existing differences in gene expression levels between the relapsed patients and patients with lasting remissions, as well as drug-induced alterations at different relapse stages compared to diagnosis. Each of the relapsed patients displayed unique chemoresistance mechanisms following similar treatment protocols, which could have been missed in a large study aimed at identifying common drug resistance determinants.

**Conclusions:**

Our findings emphasize the need for standardized evaluation of key drug transport and metabolism genes as an integral component of routine AML management, thereby allowing for the selection of treatments of choice for individual patients. This approach could facilitate the design of efficacious personalized treatment regimens, thereby reducing relapse rates of therapy refractory disease.

## Background

Acute myeloid leukemia (AML) is a heterogeneous disease originating from early precursors of the myeloid hematopoietic cell lineage [1, 2]. While no new drugs were approved for AML treatment in almost 50 years, the FDA granted approval to eight novel agents for various AML indications in the last three years [3]. However, some of these agents target specific mutations that present only in a limited subset of AML patients, and traditional intensive protocols remain the therapy of choice for most AML patients [4]. Moreover, even with this torrent of new therapeutic agents, prognosis of AML patients remains dismal with 5-year survival rates < 30% [5, 6]. The leading cause of AML-related mortality remains treatment failure due to refractory or relapsed disease, resulting from chemotherapy resistance [7]. Standard AML induction regimens mainly comprise of cytarabine (cytosine arabinoside, Ara-C) and daunorubicin (DNR), followed by high dose cytarabine consolidation. Common treatment protocols for relapsed AML may include mitoxantrone (MX) or fludarabine and etoposide (VP-16) [8]. However, in most cases high dose cytarabine is also administered. Since the efficacy of these cytotoxic drugs relies on their interaction with specific intracellular targets (Table 1 and Additional file 1: Figure S1), many drug resistance mechanisms emerge which are associated with alterations in drug transport and metabolism. These include, for example, decreased cellular accumulation due to impaired drug uptake and/or enhanced drug efflux predominantly via ATP-binding cassette (ABC) efflux transporters [9–17], loss of metabolic activation of a prodrug [11, 17–23], enhanced drug degradation [17, 24], qualitative and quantitative alterations in the target enzymes [25–28], as well as drug sequestration [29].

**Table 1:** Properties of the chemotherapeutic drugs used in AML

Drug	Target	Influx	Metabolism^a^	Efflux	Refs.
Cytarabine (Ara-C)	DNA polymerases	ENT1, CNT3, OCTN1	Activation: dCK, dCMPK, NDK. Inactivation: CDA, dCMPD, PN-I.	MRP4,7,8	[14, 30–33, 78–80]
Daunorubicin (DNR)	DNA, Topoisomerase II	Passive diffusion		P-gp, MRP1,7, BCRP	[44, 51, 81–84]
Mitoxantrone (MX)	DNA, Topoisomerase II	Passive diffusion		P-gp, MRP1, BCRP	[44, 85–90]
Etoposide (VP-16)	Topoisomerase II	Passive diffusion		P-gp, MRP1-3,6, BCRP	[16, 91, 92]
Methotrexate (MTX)	DHFR, TS, AICARFT	RFC, PCFT	Aldehyde oxidase, FPGS (polyglutamylation)	P-gp, MRP1-5, BCRP	[16, 93, 94]
Venetoclax (VEN)	Bcl-2	Passive diffusion		P-gp	[72, 95]
Gemtuzumab Ozogamicin (GO)	DNA	Ab-mediated endocytosis	Lysosomal Calicheamicin cleavage from Ab, glutathione	P-gp, MRP1	[73, 77]

DHFR, dihydrofolate reductase; TS, thymidylate synthase; AICARFT, aminoimidazole-4-carboxamide ribonucleotide formyltransferase; Bcl-2, B-cell leukemia/lymphoma 2; ENT1, equilibrative nucleoside transporter 1; CNT3, concentrative nucleoside transporter 3; OCTN1, organic cation transporter, novel, type 1; RFC, reduced folate carrier; PCFT, proton coupled folate transporter; dCK, deoxycytidine kinase; dCMPK, deoxycytidylate kinase; NDK, nucleoside diphosphate kinase; CDA, cytidine deaminase; dCMPD, deoxycytidylate deaminase; PN-I, cytosolic 5-nuleotidase 3A; FPGS, folylpoly-ɣ-glutamate synthetase; MRP, multidrug resistance-associated protein; P-gp, P-glycoprotein; BCRP, breast cancer resistance protein.

^a^Occuring in leukemic cells.

Ara-C is a nucleoside analogue pro-drug, whose active metabolite Ara-CTP blocks DNA polymerases, hence disrupting DNA replication [30]. Ara-C can be taken up into cells via several transport systems including the equilibrative nucleoside transporter 1 (ENT1, SLC29A1) [13], the concentrative Na^+^-nucleoside cotransporter 3 (CNT3, SLC28A3) [31], and the organic cation transporter, novel, type 1 (OCTN1, SLC22A4) [32]. Thereafter, Ara-C is sequentially phosphorylated to Ara-CTP by deoxycytidine kinase (dCK), deoxycytidylate kinase (dCMPK), and finally by nucleotide diphosphate kinase (NDK) [30]. Accordingly, commonly reported Ara-C resistance mechanisms include downregulation of ENT1 [11, 33–35] or CNT3 [36], loss of function of dCK [21, 34, 35, 37–42], or upregulation of the catabolic enzymes cytidine deaminase (CDA) and deoxycytidine monophosphate deaminase (dCMPD) [17, 24, 43]. Unlike other chemotherapeutic agents, enhanced Ara-C efflux is not an established mechanism of drug resistance.

DNR, MX and VP-16 are amphipathic topoisomerase II inhibitors which enter the cell via passive diffusion using a membrane flip-flop mechanism [44]. Therefore, the main determinants affecting DNR, MX or VP-16 resistance are genetic alterations in their target enzymes [25–27, 45–48] as well as increased activity of their efflux transporters, mainly P-glycoprotein (P-gp, ABCB1) [27, 47, 49–54]. Due to their lysosomotropic nature (i.e. hydrophobic weak bases), an established mechanism of resistance to all three drugs is lysosomal sequestration [55–57] which entraps these drugs away from their nuclear targets and enhances their extrusion from the cell via lysosomal exocytosis [58].

Apart from these modalities, cells may also acquire multidrug resistance (MDR) to various cytotoxic agents by acquiring resistance to apoptosis [59–63]. In this respect, a recent important addition to the armamentarium of AML treatment protocols is venetoclax (VEN), an inhibitor of the apoptosis regulator Bcl-2 [63]. VEN is currently administered in the initial induction in combination with hypomethylating agents in patients which are unfit for intensive chemotherapy [64, 65]; this combination is also effective in relapsed AML [66]. Clinical trials are underway to assess the combination of VEN with intensive chemotherapy. Resistance to VEN might result from an increase in other anti-apoptotic proteins, including the induced myeloid leukemia cell differentiation protein Mcl-1 or Bcl-XL [67–70], loss of the pro-apoptotic BAX [71], and possibly drug efflux via P-gp [72]. While VEN was initially discovered and used in distinct types of hematological cancers, other novel treatments were designed to specifically target AML [4]. In this respect, gemtuzumab ozogamicin (GO, Mylotarg) is an antibody-drug conjugate comprising an α-CD33 monoclonal antibody and a derivative of the anti-tumor antibiotic calicheamicin-γ1 [73, 74]. This conjugate enters cells via receptor-mediated endocytosis; thereafter, calicheamicin is cleaved from the antibody in the acidic lysosomal lumen and diffuses to the nucleus where it binds to DNA and inflicts strand scission [75]. GO treatment is effective in some relapsed AML patients, but treatment failure and subsequent relapse pose formidable obstacles [73, 74, 76], as GO is a substrate of the MDR efflux transporters P-gp and multidrug resistance-associated protein 1 (MRP1, ABCC1) [74, 76, 77].

The current paper focuses on two young AML patients who relapsed following chemotherapy and hematopoietic stem cell transplantation (HSCT). The paper describes their treatment course and clinical responses, and pin-points the molecular mechanisms underlying chemoresistance. Evaluation of the patients’ mRNA expression levels of specific drug transport, drug metabolism, nucleotide biosynthesis, and apoptosis genes at different retrospective stages of their disease, revealed pre-existing alterations compared to AML patients displaying lasting remissions following the same induction chemotherapy protocol. This study also uncovered alterations at the relapse stages when compared to diagnosis, which plausibly conferred drug resistance. These findings emphasize the need for standardized evaluation of key drug transport and metabolism genes as part of the routine AML management, in order to design personalized treatment regimens, thereby reducing the emergence of relapsed and/or refractory disease.

## Methods

### Patient specimens

**Fig. 1:**
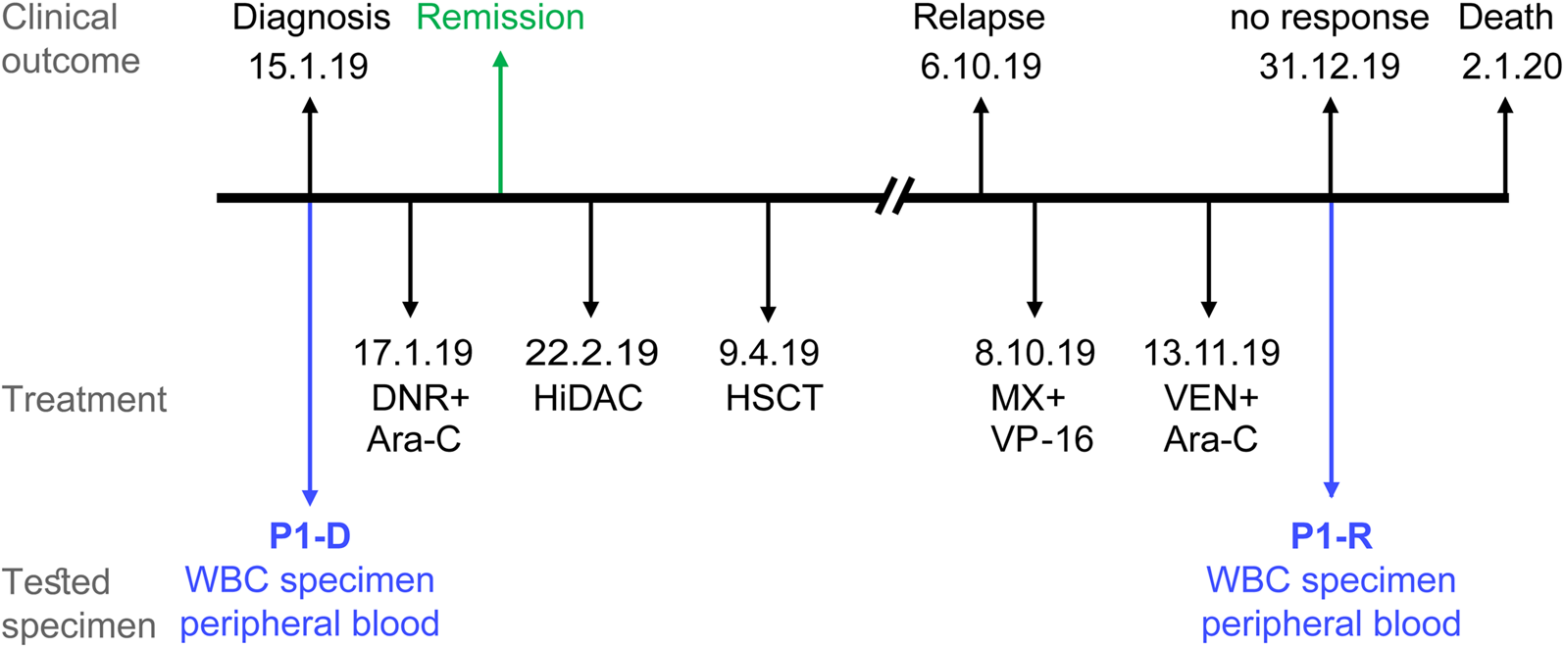
Patient 1 timeline. Depiction of all treatment courses and subsequent outcomes of patient 1, showing the stages from which specimens were obtained for analysis. Abbreviations: DNR, daunorubicin; Ara-C, Cytarabine; HiDAC, high dose Ara-C; HSCT, hematopoietic stem cell transplantation; MX, mitoxantrone; VP-16, etoposide; VEN, venetoclax

Adult AML patient specimens studied in the current paper were derived as part of the routine clinical management at the Rambam Health Care Campus (Haifa, Israel). The use of the samples was approved by the IRB committee (study number RMB 076-15) following informed consent by the patients in accordance with the Declaration of Helsinki. White blood cells (WBC) were isolated from peripheral blood or from bone marrow aspiration by Lymphoprep (STEMCELL Technologies, Vancouver, Canada) density gradient centrifugation. The resultant WBC were processed immediately for RNA isolation, or cryopreserved in RPMI-1640 medium (Gibco, Life Technologies, Grand Isle, NY) containing 40% fetal bovine serum and 10% DMSO until analysis. The gene expression levels in the subjects of this report (i.e. P1 and P2, Table 2 and Fig. 1 and 7, respectively) were compared to five bone marrow specimens from “good response” (GR) AML patients, i.e. GR1-5, displaying durable remissions following drug treatment with the same induction protocol (Table 2). Notably, the control patients were either of different age or sex compared to P1 and P2, which could be a limitation; however, we focused on comparing genes that directly impact the treatment outcome of specific drugs, regardless of patient age or sex.

**Table 2:** Patient details

	Age	Sex	Diagnosis	Karyotype/Mutations	ELN risk score	WHO AML category	De-Novo/secondary AML	WBC at diagnosis	Extramedullary involvement
P1	27	Female	Jan 2019	T (10;11) (p11;q11), del (9) (q13q22) [3]/ Normal [17]	Intermediate	M4 - acute monoblastic/ monocytic leukemia	*De novo*	1.24 × 1000/mcl	No
P2	18	Female	Dec 2017	t(8;21)	Favorable	M1- AML without maturation	*De novo*	3.05 × 1000/mcl	No
GR1	38	Male	May 2016	Normal Karyotype, NPM1^mut^	Favorable	M2- AML with maturation	*De novo*	166 × 1000/mcl	No
GR2	62	Male	Sep 2018	Normal Karyotype, NPM1^mut^	Favorable	M1- AML without maturation	*De novo*	6.6 × 1000/mcl	No
GR3	69	Male	Jan 2017	Normal Karyotype	Intermediate	M2- AML with maturation	*De novo*	11.7 × 1000/mcl	No
GR4	63	Female	Dec 2010	Normal Karyotype	Intermediate	M1- AML without maturation	*De novo*	4.4 × 1000/mcl	No
GR5	28	Male	Oct 2013	t(8;21)	Favorable	M2- AML with maturation	*De novo*	25.8 × 1000/mcl	No

### RNA purification and cDNA synthesis

WBC were centrifuged at 800xg for 3 min and washed twice with PBS. RNA was isolated using TRI Reagent according to the instructions of the manufacturer (Sigma Aldrich, St. Louis, MO, USA). One µg RNA was used for cDNA synthesis using the high capacity cDNA reverse transcription kit according to the manufacturer’s instructions (Thermo Fisher Scientific, Waltham, MA, USA).

### Quantitative Real-Time (RT) polymerase chain reaction (PCR)

RT-PCR was performed in triplicates with the perfeCTa SYBR Green SuperMix (Quanta bio, Beverly, MA, USA) using 150 nM forward and reverse oligonucleotide primers (Table 3) and 0.25 ng/µl cDNA per reaction. Gene expression levels were normalized to glucuronidase β (GUSB) that was used as an internal control. RT-PCR reactions were performed using the 7300 Real-Time PCR System, and results were analyzed with the 7300-system sequence detection software version 1.4 (Applied Biosystems, CA, USA).

**Table 3:** RT-PCR primers

Gene	Forward primer sequence	Reverse primer sequence	Refs.	Refseq accession
ENT1	GGGCAGCCTGTTTGGTCT	CCACTGGCAATAGCGCAG	[96]	NM_001078177.2
ENT2	CTCCTGTCCATGGCCAGTG	GGGCCTGGGATGATTTATTG	[96]	NM_001300868.1
ENT3	TCAGCGGTGCCTCCACTGT	GCAGCCAAGTCCACCAATGA	[96]	NM_018344.6
CNT3	ACATTTCTTTTGGGGTTCCAT	GCAATCAGATTCACAGCGATG	[96]	NM_001199633.2
dCK	GCCGCCACAAGACTAAGGAA	GACTTCCCTGCAGCGATGTT	[42]	NM_000788.3
CDA	TGTGCTGAACGGACCGCTA	GCAGGTCCTCAGGCCCAA	[42]	NM_001785
NDK	ATTCCGCCTTGTTGGTCTGA	TTGGAGTCTGCAGGGTTGGT	Current	NM_198175
PN-I	AACAACATAGCATCCCCGTGT	TTCCTCAAGGCACCATCATGT	Current	NM_001002010.5
BCRP	GGATGAGCCTACAACTGGCTT	CTTCCTGAGGCCAATAAGGTG	[97]	NM_004827.3
MRP1	GTGTTTCTGGTCAGCCCAACT	TTGGATCTCAGGATGGCTAGG	[97]	NM_004996
P-gp	CCGACTTACAGATGATGTCTCCAA	CAGACAGCAGCTGACAGTCCAA	Current	NM_000927
BCL2	GTCATGTGTGTGGAGAGCGTCA	GGCAGGCATGTTGACTTCACTT	Current	NM_000633
BCLX(L)	TCTTCCGGGATGGGGTAAAC	AAGCGTTCCTGGCCCTTTC	Current	NM_138578
MCL1	GGACAAAACGGGACTGGCTAG	TGGCTAGGTTGCTAGGGTGC	Current	NM_021960
CTSD	TGCTCAAGAACTACATGGACGC	CGAAGACGACTGTGAAGCACT	[98]	NM_001909
ATP6V1H	AGCCCTGAAGAGAAGCAAGAGA	CGATTCAACATTGGCAGAAAGT	[98]	NM_015941
MSMO1	AGCATCCTTGGCTGTGGAATAT	CCCATGTCTCTGGCTTATCCTT	Current	NM_006745
HMGCR	GGGAAAATATTGCTCGTGGAAT	CAAGGACACACAAGCTGGGAA	Current	NM_000859.3
CAD	GGTCTCTGCAAGTGGTTTGAA	CCTGTTCCCGCAACTTCTT	[96]	NM_004341
CTPS	CCCCAGATCTGGTTGTATGCA	AAGCGATCATATCTGTCAGCCA	Current	NM_001905
UMPS	GGATTATGGAACTAAGCGTCTTGT	CACACTGAGTGGAGGCGGAT	Current	NM_000373
GART	GTGGAGGAAGGGAACATACGC	TCTCTTTGCAGAATTGAGCAAGG	[99]	NM_000819
TS	TCCCGAGACTTTTTGGACAGC	TGATGGTGTCAATCACTCTTTGC	[99]	NM_001071
DHFR	ATGCCTTAAAACTTACTGAACAACCA	TGGGTGATTCATGGCTTCCT	[99]	NM_000791
RFC	ACCATCATCACTTTCATTGTCTC	ATGGACAGGATCAGGAAGTACA	[99]	NM_194255
FPGS	GAGAGGCCGAGCGGTGG	TGCCTGTGGATGACACCTCTG	Current	NM_004957
GUSB	CCATTCCTATGCCATCGTG	ATGTCGGCCTCGAAGGG	[96]	NM_000181

### Correlation of gene expression and overall survival in AML

Correlation between gene expression levels and overall survival (OS) in AML patients was calculated and plotted using the *GEPIA2* server [100]. The analysis was set to quartile cutoff and gene expression levels were normalized to DNA-directed RNA polymerase I subunit D (POLR1D).

## Results and discussion

### Patient 1

Patient 1 (P1), which was diagnosed with AML on January 15th, 2019 (Fig. 1, Table 2), presented with a karyotype containing the translocation t (10;11)(p11;q11) and the deletion del(9)(q13q22) in 15% (3/20) of cells. She received standard induction chemotherapy of Ara-C (7 days, 100 mg/m^2^) and DNR (3 days, 90 mg/m^2^) which resulted in remission. This was followed by consolidation with high dose Ara-C (HiDAC, 3 g/m^2^, 6 doses). The patient underwent allogeneic HSCT while in complete remission (CR) 1, but relapsed six months later. A salvage protocol of MX (2 days, 30 mg/m^2^) and VP-16 (5 days, 100 mg/m^2^) was administrated with no response. Thus, subcutaneous low dose Ara-C (10 days, 20 mg/m^2^) and VEN (1 day, 600 mg) were prescribed. Following treatment, the patient suffered from severe leukopenia, developed sepsis and succumbed to her disease within less than a year from diagnosis.

**Fig. 2:**
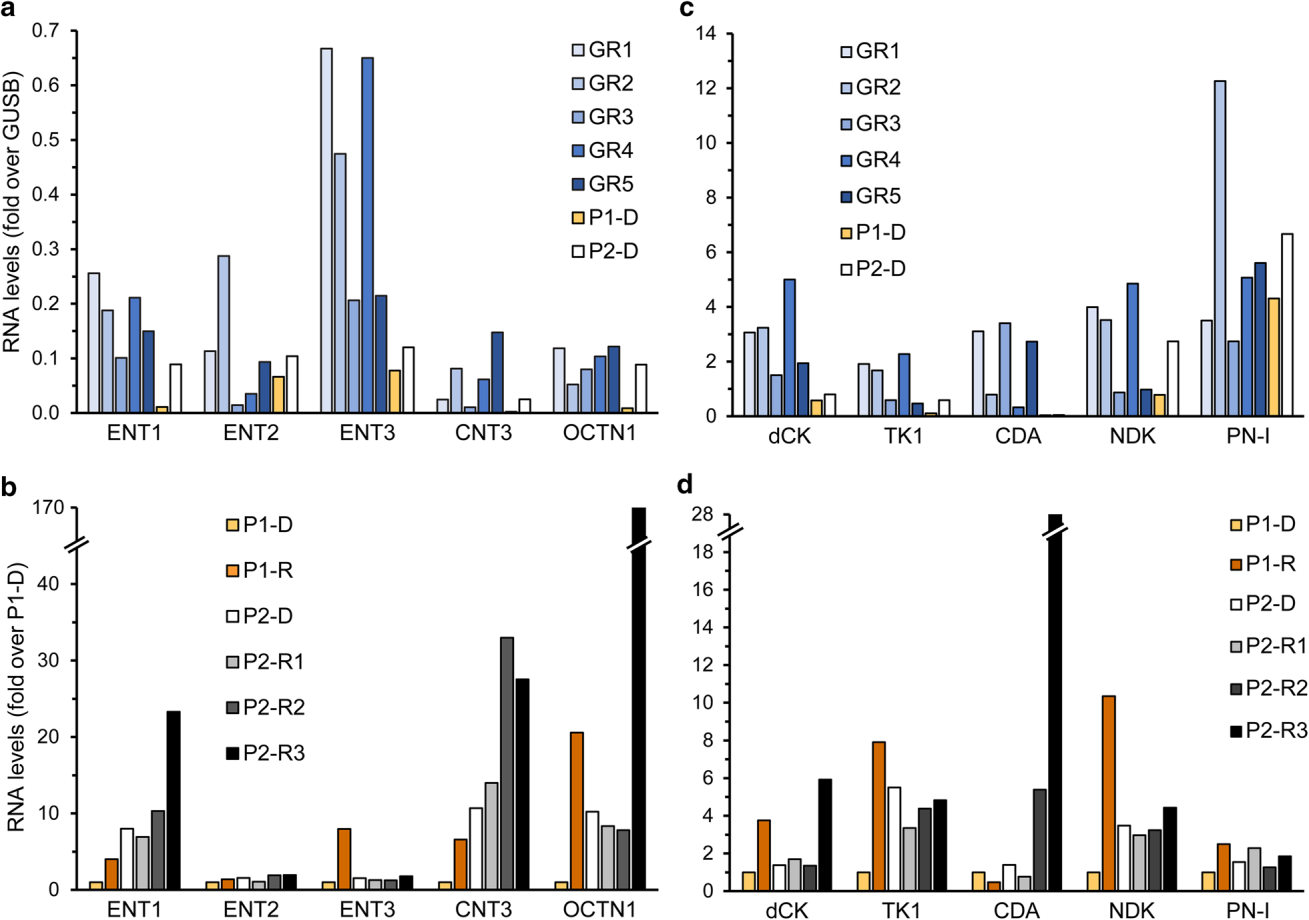
Expression levels of nucleoside influx transporters and nucleoside salvage pathway enzymes. RNA was purified from AML patient specimens using tri reagent as described in the Methods section. Gene expression levels were determined using quantitative RT-PCR. **a**,** b** Comparison of the expression levels at diagnosis in patients with good response to chemotherapy (GR1-5) and in relapsed patients (P1-D and P2-D). The results are presented as fold over GUSB which served as an internal control. **c**, **d** Comparison of the expression levels at the diagnosis and relapse stages in the relapsed patients. Results shown are normalized to GUSB which served as an internal control, and presented as fold over the expression levels of P1-D

**Fig. 3:**
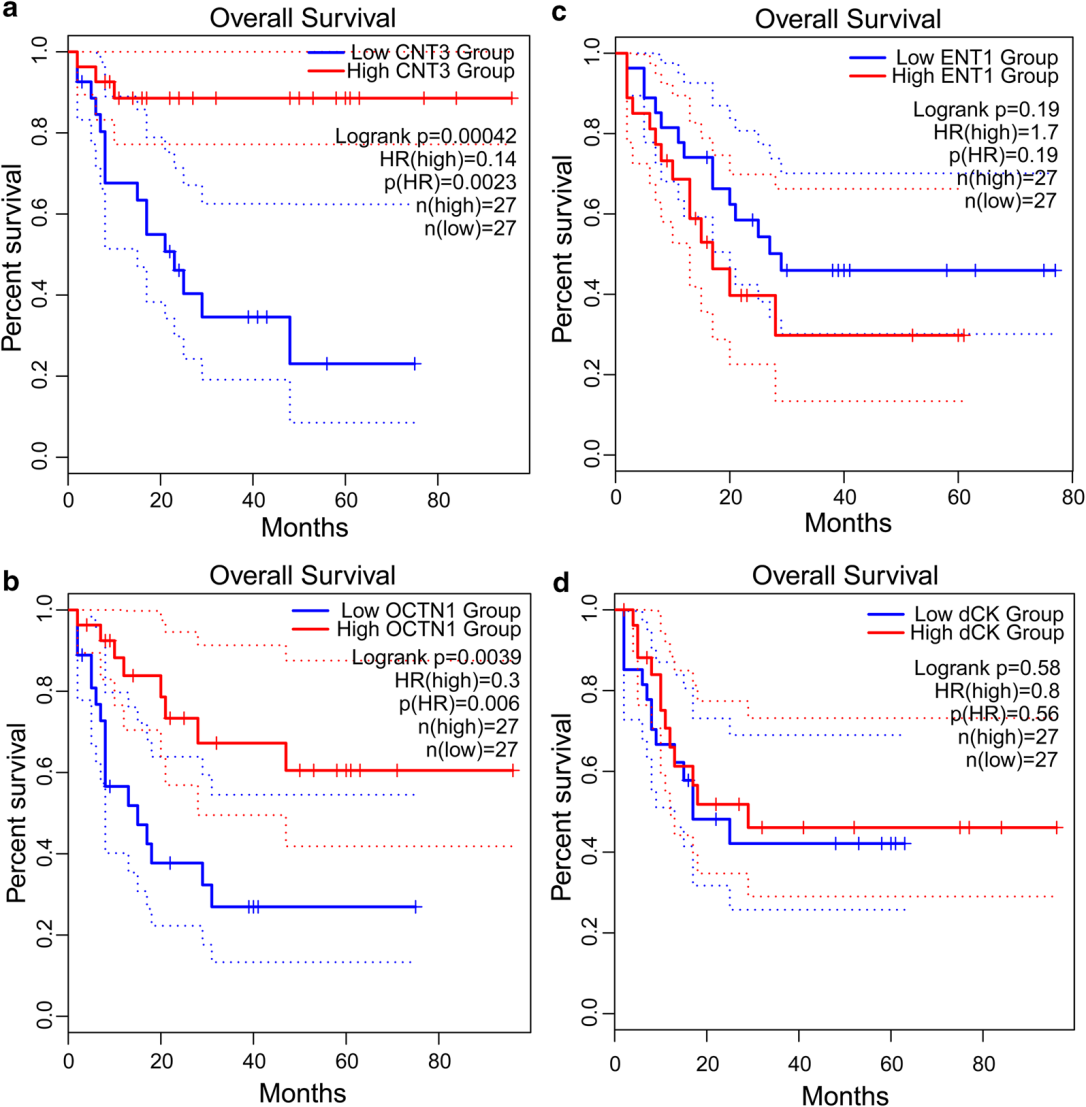
Correlation of gene expression levels with overall survival in AML. The GEPIA2 server was used to generate correlation analyses between gene expression levels in AML bone marrow specimens from the cancer genome atlas (TCGA) and overall patient survival. Gene expression levels were normalized to POLR1D

**Fig. 4:**
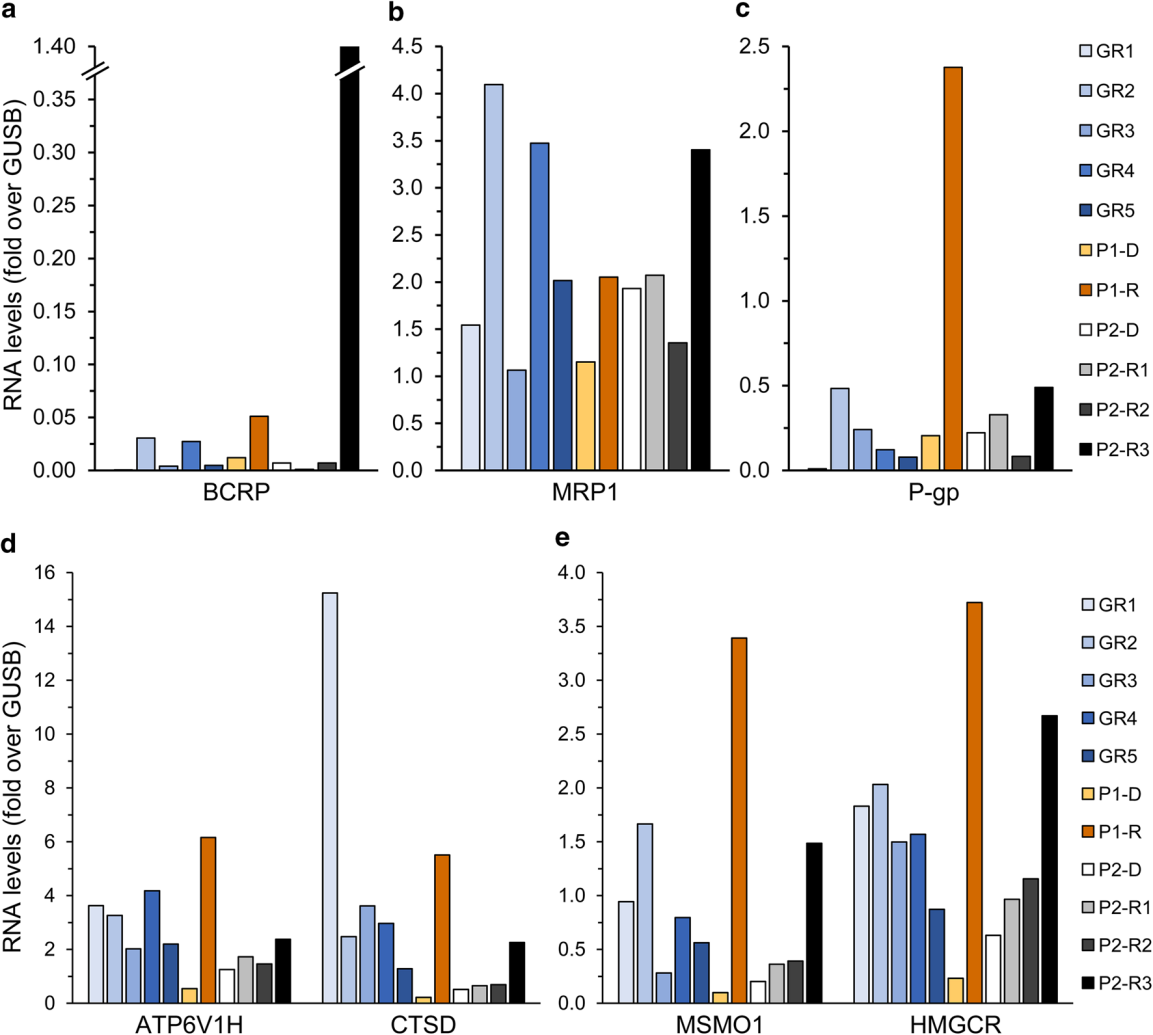
RNA expression of drug efflux transporters, lysosomal proteins and cholesterol biosynthesis enzymes. RNA was purified from AML patient specimens using tri reagent, and gene expression levels were evaluated using quantitative RT-PCR, as described in the Methods section. Gene expression levels in relapsed patients (P1 and P2) at the different stages of their disease were compared to those in patients with good response to chemotherapy (GR1-5) at diagnosis. **a**–**c** Multidrug resistance efflux transporters, **d** Lysosomal proteins, and **e** Cholesterol biosynthesis enzymes. The results are displayed as fold over the internal control, GUSB

To identify the molecular mechanisms underlying treatment failure in P1, we performed a retrospective gene expression analysis on two WBC specimens from this patient: one from the time of diagnosis (i.e. 15.1.19, P1-D) and another post-treatment sample after the relapse (i.e. 31.12.19, P1-R). At diagnosis, P1 displayed silencing of the Ara-C influx transporters ENT1, CNT3 and OCTN1 (Fig. 2a), as well downregulation of dCK, the rate-limiting enzyme in Ara-C pro-drug activation (Fig. 2c), compared to GR patients. This indicated a major impairment of both Ara-C uptake and bioactivation in P1, which would severely hinder its cytotoxic activity. Consistently, survival analysis using the GEPIA2 server revealed significant correlations between low expression levels of either CNT3 or OCTN1 and poor prognosis in AML (i.e. short OS, Fig. 3a, b); however, no such correlation was observed for low levels of ENT1 or dCK (Fig. 3c, d). Since dCK is absolutely required for Ara-C activation, a possible reason that dCK levels were not correlated to any specific disease outcome could be due to high expression of an inactive enzyme, i.e. through alternative splicing [21, 101, 102], which might interfere with this analysis. The correlation between low OCTN1 expression and poor survival in AML is further supported by a previous study on pediatric AML patients, which established OCTN1 as an Ara-C influx transporter and showcased low OCTN1 expression as a predictor of poor survival in AML patients [32]. In the same study, ENT1 also displayed a similar, albeit less significant correlation; this trend was previously reported [12]. Regarding CNT3 silencing, we note that the CNT3 gene locus maps to chr9q21, which resides within the deleted region found in 15% of the blasts in P1; this is a well-established and frequently deleted region in AML patients [103–105]. Considering these findings, Ara-C presumably had a minimal cytotoxic effect in the blasts of P1, rendering its use in the induction and consolidation phases rather futile. We hence propose that patients could benefit from evaluating the gene expression status of Ara-C influx transporters prior to treatment, in order to ensure tumor cell accumulation of Ara-C especially in the presence of genomic deletions of the CNT3 locus. Although Ara-C might not have been effective, the induction treatment did induce remission in P1 presumably due to DNR cytotoxicity, since neither of the MDR efflux transporters, P-gp and BCRP, were substantially expressed at diagnosis (Fig. 4a, b).

Consistent with the downfalls of monotherapy [106], i.e. DNR as a single agent, P1 relapsed within nine months of DNR treatment. We therefore explored possible changes in gene expression that could have rendered the relapsed disease resistant to further drug treatment. The notably elevated P-gp levels at relapse (nearly 12-fold over diagnosis, Fig. 4b) suggested clonal expansion of a P-gp-dependent DNR-resistant clone, which presumably led to VP-16 and MX resistance [16, 47, 107]. Along this vein, since DNR is a lysosomotropic drug (LD) it might have induced an expansion of the lysosomal compartment [55, 56], rendering the cells resistant to other hydrophobic weak base drugs such as MX and VP-16 [29, 55, 108–110]. To explore the possible contribution of lysosomes to the chemoresistance that emerged at relapse, we tested the levels of two genes from the coordinated lysosomal expression and regulation (CLEAR) network [111, 112], which is upregulated upon drug-induced lysosomal biogenesis [55, 98]. We found a major upregulation of both the lysosomal acidification pump V-type proton ATPase subunit H (ATP6v1H, 11-fold, Fig. 4d) and the acidic lysosomal protease cathepsin D (CTSD, 25-fold, Fig. 4d) [111], which indicated a stable expansion of the lysosomal compartment. To corroborate lysosomal drug sequestration at relapse, we chose representative genes that are dramatically elevated upon lysosomal dysfunction following treatment with LDs. Various LDs have been shown to hinder the export of cholesterol from lysosomes, where cholesterol accumulates [113–116], leading to enhanced expression of mevalonate pathway genes in an attempt to compensate for the low cellular cholesterol levels [114, 115]. Indeed, the gene expression levels of the cholesterol biosynthesis enzymes methylsterol monooxygenase 1 (MSMO1) and 3-hydroxy-3-methylglutaryl-coenzyme A reductase (HMGCR) were dramatically increased post relapse (i.e. 34- and 16-fold over diagnosis, respectively, Fig. 4e).

**Fig. 5:**
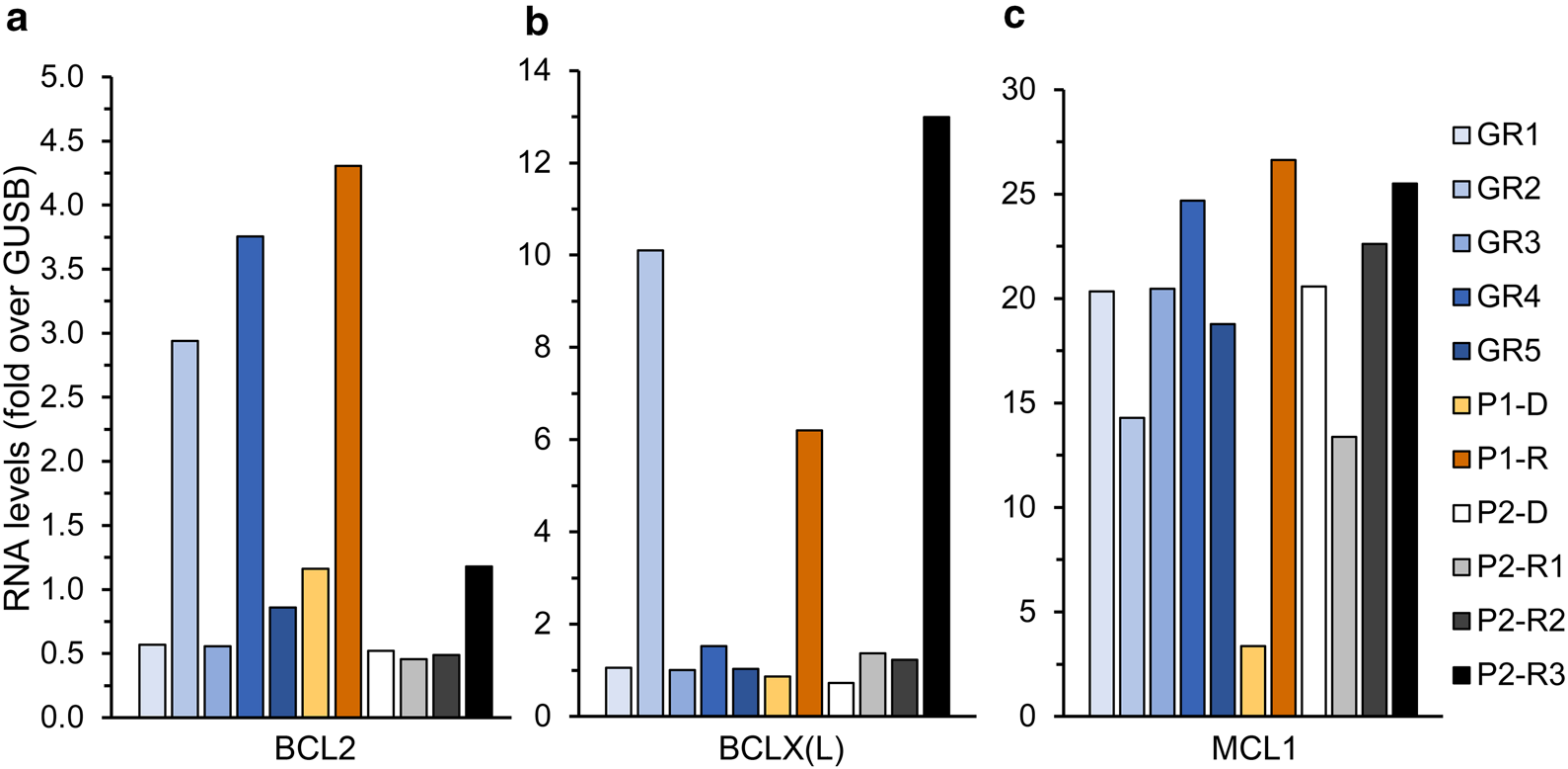
RNA expression of genes encoding for anti-apoptotic proteins. RNA was purified from AML patient specimens using tri reagent, and gene expression levels were quantified using RT-PCR as described in the Methods section. The expression levels of Bcl-2 (**a**), Bcl-XL (**b**), and Mcl-1 (**c**) in relapsed patients (P1 and P2) at different stages of their disease, were compared to those in patients with good response to chemotherapy (GR1-5) at diagnosis. The results are presented as fold over the internal control, GUSB

Increased expression levels of the anti-apoptotic genes BCL2, BCLX(L) and MCL1 post-relapse (~4-, 7-, and 8-fold over diagnosis, respectively, Fig. 5) indicated an aggressive anti-apoptotic AML phenotype underlying resistance to VEN, as was previously reported [67, 68, 70].

Alongside the inhibition of apoptosis, P1-R displayed enhanced expression of nucleoside salvage and biosynthesis genes that could support enhanced DNA replication. The activated nucleoside salvage pathway included the nucleoside influx transporters ENT3 and OCTN1 (8- and 20-fold increase, respectively, Fig. 2b), and the nucleoside kinases dCK, TK1 and NDK (4-, 8-, and 10-fold, respectively, Fig. 2d). Additionally, there was a 2.5-fold increase in the gene expression levels of cytosolic 5'-nucleotidase 3A (cN-III/PN-I, Fig. 2d), that could dephosphorylate Ara-CMP and counteract its cytotoxic activity [117–120], which might have otherwise been increased upon upregulation of OCTN1, dCK and NDK.

**Fig. 6:**
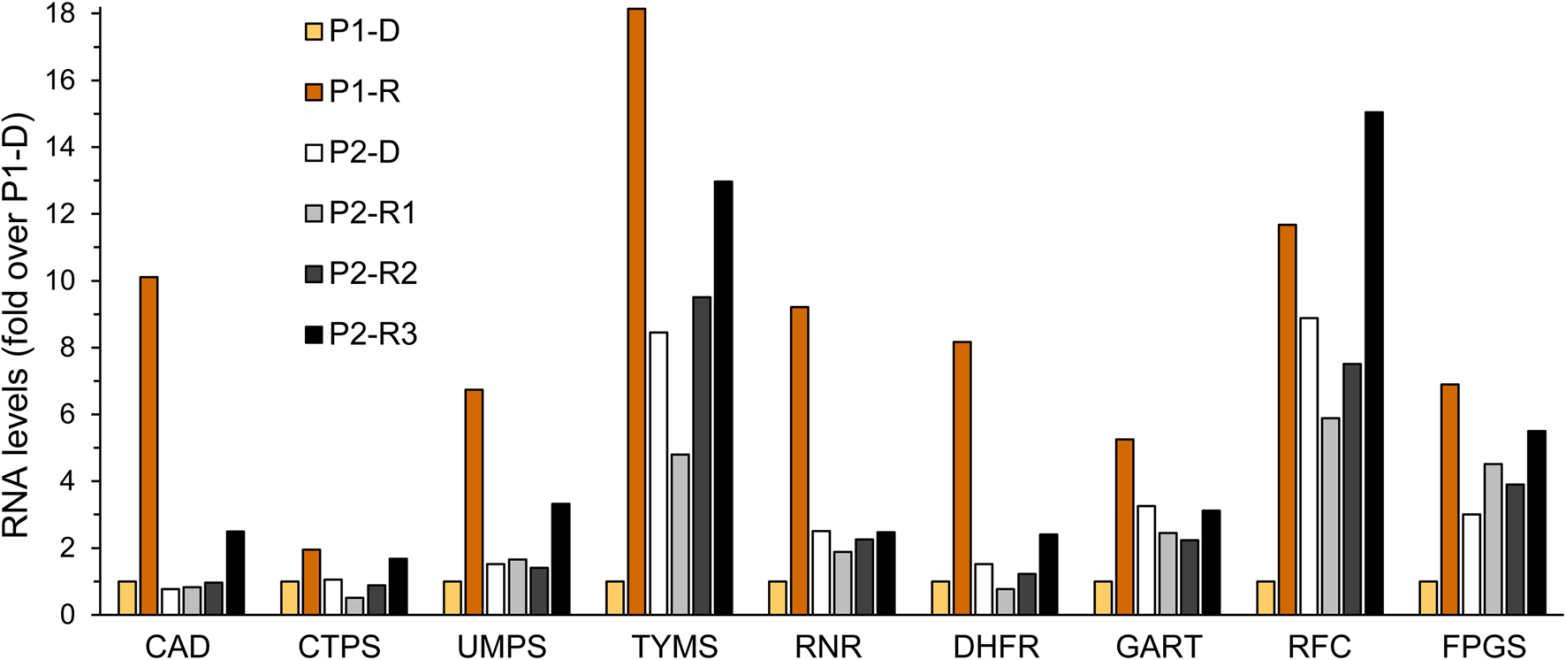
RNA expression of *de novo* nucleotide biosynthesis pathway (DNSP) and folate metabolism genes. RNA was purified from AML patient specimens using tri reagent as described in the Methods section. Gene expression levels were evaluated using quantitative RT-PCR in relapsed patients (P1 and P2) at the different stages of their disease. The results are normalized to GUSB which served as an internal control, and presented as fold over P1-D

In addition to the nucleoside salvage pathway, the *de novo* nucleotide synthesis pathway (DNSP) was also significantly activated following drug treatment, along with the relevant folate metabolism genes (Fig. 6 & Additional file 1: Figs. S2–S4). This entailed upregulation of the mRNA levels of all genes studied including: the trifunctional CAD enzyme (CAD, 10-fold), CTP synthase 1 (CTPS1, 2-fold), uridine 5'-monophosphate synthase (UMPS, 7-fold), thymidylate synthase (TYMS, 18-fold), ribonucleoside-diphosphate reductase subunit M1 (RRM1, 9-fold), dihydrofolate reductase (DHFR, 8-fold), phosphoribosylglycinamide formyltransferase (GART, Trifunctional purine biosynthetic protein adenosine-3, 5-fold), reduced folate carrier (RFC, SLC19A1, 12-fold), and folylpoly-γ-glutamate synthetase (FPGS, 7-fold). Apart from enabling enhanced DNA replication, upregulation of DNSP genes can lead to an expansion of the cellular nucleotide pools including dCTP, which might further competitively negate Ara-C cytotoxicity [121]. In this respect, upregulation of RRM1 can lead to increased cellular dCTP levels, thereby inhibiting dCK-mediated Ara-C activation and blocking Ara-CTP incorporation into DNA [43].

Performing an analysis, such as the one presented here, prior to the treatment decision might have revealed a superior personalized treatment modality, for example by targeting the DNSP using a combination of azidothymidine (AZT) and hydroxyurea (HU) as we have recently demonstrated [42]. This plausible treatment modality could be specifically viable in light of the upregulation of TK1 (Fig. 2d), which could lead to enhanced AZT activation [122–124]. While AZTMP is a substrate of the inactivating PN-I, its *K*_m_ value is 120-fold higher than that of CMP [118], and to date, there have been no reports of PN-I-mediated AZT resistance.

### Patient 2

**Fig. 7:**
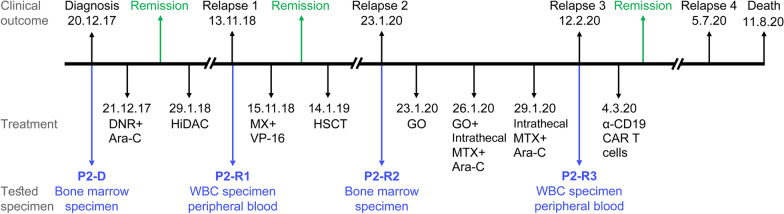
Patient 2 timeline. Depiction of all treatment courses and subsequent outcomes of patient 2, showing the stages from which specimens were derived for analysis. Abbreviations: DNR, daunorubicin; Ara-C, Cytarabine; HiDAC, high-dose Ara-C; MX, mitoxantrone; VP-16, etoposide; HSCT, hematopoietic stem cell transplantation; GO, gemtuzumab ozogamicin; MTX, methotrexate; CAR, chimeric antigen receptor

P2 was diagnosed with AML on December 20th, 2017 (Fig. 7, Table 2), and harbored the recurrent translocation t (8;21), considered as the most favorable cytogenetic abnormality [125]. She underwent remission following standard Ara-C and DNR induction therapy, and remained disease-free for eleven months after consolidation treatment with HiDAC. Gene expression analysis on the diagnosis sample of P2 (20.12.17, P2-D) revealed levels of ENT1, CNT3 and OCTN1 comparable to those of the GR patients (Fig. 2a), indicating sufficient Ara-C influx; and while dCK levels were relatively low (Fig. 2c), NDK levels were high and the Ara-C catabolic enzyme CDA was not expressed (Fig. 2c), suggesting sufficient Ara-C activation. Moreover, the low expression levels of BCRP and P-gp along with comparable levels of MRP1 (Fig. 4a–c) supported the cellular accumulation of DNR. However, despite these apparently positive characteristics, relapse occurred.

At the first relapse (13.11.18, P2-R1), within the scope of genes we studied, there was no evidence of pertinent gene expression alterations that might directly underlie or lead to chemotherapy resistance. There was a moderate increase in the RNA levels of the anti-apoptotic protein Bcl-XL (1.9-fold relative to diagnosis level, Fig. 5b), albeit not higher than in the reference GR patients. Interestingly, MSMO1 and HMGCR expression levels started to rise (1.8- and 1.5-fold relative to diagnosis levels, Fig. 4e), possibly due to the lysosomotropic activity of DNR, as discussed above. However, there was no marked LD-associated resistance, as the salvage treatment composed of MX and VP-16 led to a second remission. This was followed by an allogeneic HSCT and durable remission for another 12 months.

At the second relapse (23.1.20, P2-R2), there was a 3-fold increase in CNT3 expression levels (Fig. 2b), which could imply an attempt to enhance nucleoside salvage to support DNA replication. Moreover, there was a 4-fold increase in the transcript levels of CDA. Since this CDA increase was not observed in P2-R1, we cannot determine that it was induced by the HiDAC consolidation treatment, 13 months earlier. These changes were detrimental to the next salvage treatment of GO (3 g/m^2^, 3 doses) followed by intrathecal methotrexate (MTX, 12.5 mg) and Ara-C (30 mg), which failed to induce remission.

Three weeks after R2, when disease persistence was assessed (12.2.20, P2-R3), the patient displayed a further increase in CDA mRNA levels (~5-fold over R2, and 20-fold over diagnosis, Fig. 2d), possibly due to a rapid clonal expansion of cells detected in P2-R2. At R3, P2 exhibited multiple modes of MDR: (1) Increased CDA expression which presumably constituted the underlying basis for the recent Ara-C resistance [24]. (2) A marked 20-fold increase in BCRP mRNA levels, and a 2-fold increase in MRP1 levels (Fig. 4a, b) led to MTX-resistance, as MTX is a *bona fide* transport substrate of BCRP [15, 16] and MRP1 [15, 126]. (3) The apparent increase in MRP1 levels together with that of P-gp (Fig. 4c) might have decreased cellular GO levels, hence reducing its cytotoxicity [73, 77, 127]. (4) The anti-apoptotic profile of P2 was exacerbated, including extreme upregulation of BCLX(L) (10-fold over R2, and 18-fold over diagnosis, Fig. 5b) and > 2-fold increase in BCL2 levels (Fig. 5a); this contributed to both MTX [128, 129] and Ara-C [129] resistance. (5) The transcript levels of CTSD, MSMO1, and HMGCR were increased by 4.4-, 7.3- and 4.2-fold over diagnosis, respectively (Fig. 4d, e), indicating the expansion of the lysosomal compartment and drug sequestration; this could be relevant for the GO treatment which is metabolized in lysosomes [73]. Although further research is warranted to determine whether the GO conjugate or any of its derivatives might become sequestered within lysosomes, none of the other drugs after R2 could have triggered such a lysosomotropic response.

The continued upregulation of CNT3, and further increases in the gene expression levels of OCTN1, ENT1 (Fig. 2b) and dCK (Fig. 2d), indicated the upregulation of the nucleoside salvage pathway. The latter could have been strategically targeted with CDA-independent nucleoside analogs such as 6-mercaptopurine and 6-thioguanine [130–132], which would benefit from the increase in CNT3 levels [133].

Following the failure of the last treatment, and since P2 had CD19 positive blasts, she was able to receive CD19-targeted chimeric antigen receptor (CAR) T cell therapy [134, 135], which resulted in a short remission of three months. Recent reports on the challenges of CAR T cell therapy in general [136], and specifically in AML due to its immunosuppressive microenvironment [137, 138], might explain the short duration of remission following the last treatment. At the last relapse, P2 suffered from sepsis during the HSCT procedure and succumbed to her disease, 32 months after the first diagnosis.

**Fig. 8:**
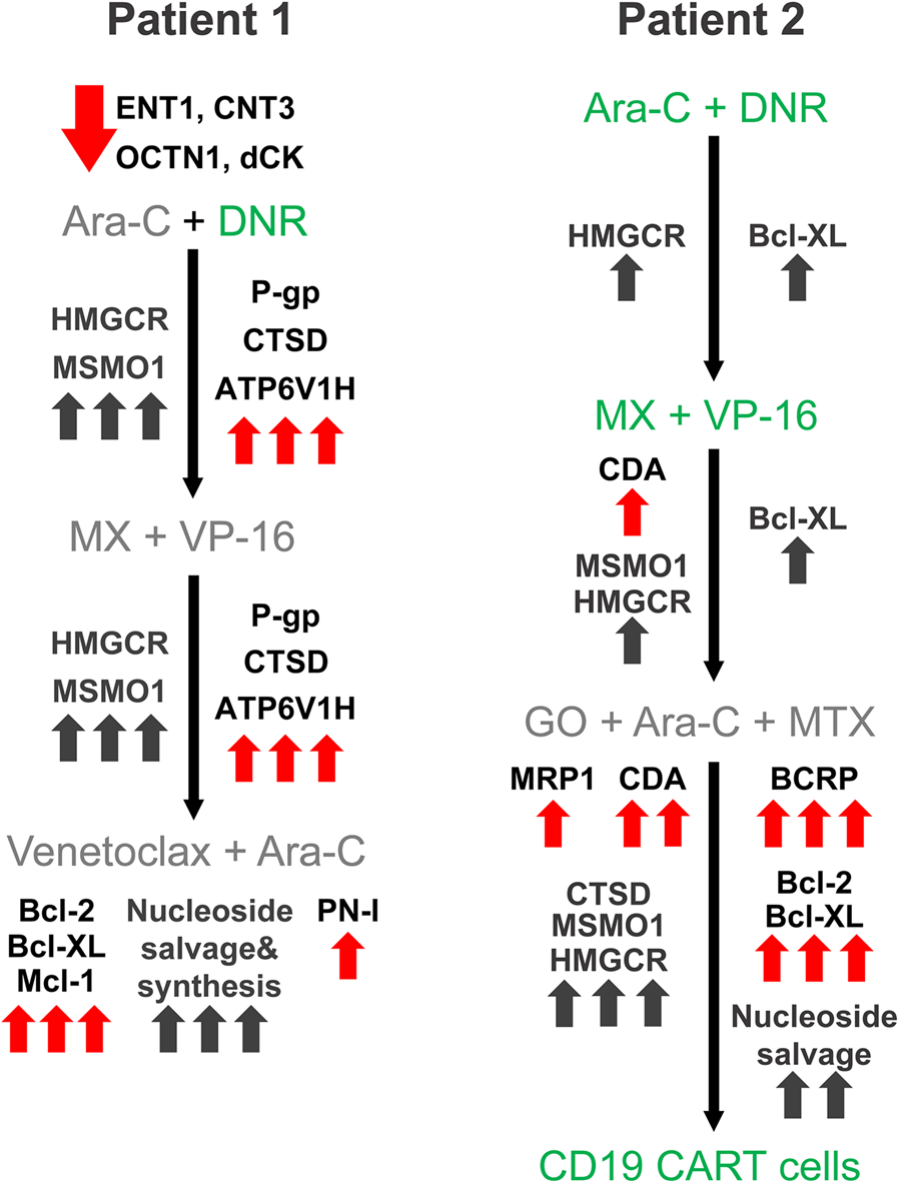
Summary of the chemotherapeutic treatments and consequent gene expression alterations. This scheme showcases the potential associations between specific drug treatments and gene expression changes which possibly conferred drug resistance and contributed to relapse. The assignment of changes to specific stages in P1 is only implied, as the relapse specimen was obtained after all of the treatments. Chemotherapeutic drugs that plausibly induced remission are denoted in green

A summary of the chemotherapy-related gene expression alterations in the two patients is depicted in Fig. 8.

The current paper focused on two complex cases of relapsed AML in young patients. Both patients were characterized by aberrant expression of genes involved in the transport and metabolism of anchor chemotherapeutic drugs for AML treatment including Ara-C, DNR, MX, VP-16 and VEN. Therefore, both patients could have possibly benefited from a continuous assessment of expression of genes mediating drug activity or resistance with the aim to tailor efficacious personalized treatment regimens. While AML is a highly heterogeneous hematological malignancy with high prevalence of treatment failure, a handful of genes are invariably relevant for treatment outcome due to their direct role in drug transport and metabolism including drug influx and efflux, prodrug bioactivation, drug targets, drug inactivation or degradation as well as drug compartmentalization away from the drug target. Therefore, the expression status of these genes is crucial for predicting the potential efficacy of specific treatments as well as assessing post-treatment response. This can allow for an optimal tailoring of chemotherapeutic regimens, thereby enhancing the achievement of long-term remissions. In this respect, special consideration should be given when selecting the drugs for the salvage treatment in relapsed disease. Since relapse usually occurs due to the expansion of resistant clones, if the salvage treatment is composed of drugs with the same characteristics (e.g. same cellular target, or influx/efflux transporters) as the induction drugs, it is likely that the same resistance mechanisms will hamper the cytotoxicity of the salvage drugs. For example, leukemic cells with intrinsic or acquired resistance to DNR either via downregulation of topoisomerase II [48, 139], upregulation of P-gp or expanded lysosomes, are likely to be cross-resistant to MX and VP-16, thereby inducing another relapse, since all of these drugs share similar cellular pathways. Similarly, leukemic cells that survived Ara-C cytotoxicity might be cross-resistant to other nucleoside analog prodrugs that share the same cellular uptake and activation routes. Consideration of drug action mechanisms and metabolism could further suggest potential combinations that may readily overcome chemoresistance modalities [42, 140–143].

The recent pervasiveness of next-generation, high-throughput techniques for sequencing, gene expression, and proteomics led to the identification of previously unpredicted genes that correlate with specific AML prognoses. However, big-data studies search for a common denominator in multiple patients and might lack molecular mechanistic insight into the role of specific gene expression alterations in disease progression and therapy response. The results of the current study, in concordance with a previous paper [144], suggest that AML patients would benefit from standardized testing of well-characterized relevant genes in order to tailor treatment regimens using a plethora of clinically available drugs. Given that conventional chemotherapy remains the cornerstone of AML treatment, and since chemoresistance continues to be the primary impediment towards curative AML treatment, real-time evaluation of drug resistance mechanisms remains a crucial task for the design of efficacious personalized AML treatments. For this to become a tangible possibility, there is a burning need to develop a standard protocol and/or a chip-based gene expression array that could be easily introduced into the clinical setting. Although, not all AML patients will benefit from such an analysis since some of the underlying resistance mechanisms may remain elusive, the prospect of improving patient survival rates along with minimization of adverse side effects inflicted by ineffective drug treatments, should be a priority of paramount importance.

## Conclusions

The formidable heterogeneity of AML calls for the development of individualized treatment strategies. The genes studied in the current paper are crucial for treatment outcome and therefore should be routinely evaluated and taken into consideration when selecting the treatment of choice for individual AML patients. These evaluations are relevant both at the time of diagnosis to assess any potential preexisting resistance modalities, as well as at relapse in order to decipher the mechanisms underlying chemoresistance. Specifically, the risk of cross-resistance should be avoided by the administration of antitumor agents with distinct modes of action at consequent disease stages. Standard gene expression testing can help physicians to refrain from employing chemotherapy that would be ineffective in a specific patient, reduce unnecessary adverse effects, and in some cases even reveal a targeted personalized treatment possibility. In summary, towards a curative treatment of individual AML patients, we herein propose that an assortment of well-defined genes contributing to chemotherapeutic drug activity and/or chemoresistance be evaluated both at diagnosis as well as throughout the entire course of the disease in order to select the treatment of choice for individual AML patients.

## Supplementary Information

**Additional file 1: Figure. S1.** Summary of drug metabolism. A graphical depiction of cellular transport, metabolism, and targets of AML chemotherapeutic drugs discussed in the manuscript. Pro-drug intermediates are colored in black, bioactive drugs are colored in light blue, and inactivated metabolites are colored in grey. Abbreviations: AICARFT, aminoimidazole-4-carboxamide ribonucleotide formyltransferase; Ara-C, cytosine arabinoside; ATP6V1H, V-type proton ATPase subunit H; Bcl-2, B-cell leukemia/lymphoma 2; Bcl-XL, apoptosis regulator Bcl-X, long isoform; BCRP, breast cancer resistance protein; CDA, cytidine deaminase; CNT3, concentrative nucleoside transporter 3; CTSD, cathepsin D; dCK, deoxycytidine kinase; dCMPD, deoxycytidylate deaminase; dCMPK, deoxycytidylate kinase; DHFR, dihydrofolate reductase; DNR, daunorubicin;ENT1, esquilibrative nucleoside transporter 1; FPGS, folylpoly-ɣ-glutamate synthetase; GO, Gemtuzumab Ozogamicin; HMGCR, 3-hydroxy-3-methylglutaryl-coenzyme A reductase; Mcl-1, Induced myeloid leukemia cell differentiation protein Mcl-1; MRP, multidrug resistance-associated protein; MSMO1, methylsterol monooxygenase 1;MTX-polyG, methotrexate polyglutamate; MX, mitoxantrone; NDK, nucleoside diphosphate kinase; OCTN1, organic cation transporter, novel, type 1; PCFT, proton coupled folate transporter; P-gp, P-glycoprotein; PN-I, cytosolic 5-nuleotidase 3A; RFC, reduced folate carrier; TS, thymidylate synthase; VEN, venetoclax; VP-16, etoposide. **Figure S2.** Biosynthetic pathway of purine nucleotides. A graphical depiction of cellular *de novo* biosynthesis of purines. The enzymes that are discussed in the manuscript are colored in red. Abbreviations: ADSL, adenylosuccinate lyase;ADSS, adenylosuccinate synthase; AK, adenylate kinase; ATIC, 5-aminoimidazole-4-carboxamide ribonucleotide formyltransferase; GART, phosphoribosylglycinamide formyltransferase;GMPS, guanine monphosphate synthase;GUK1, guanylate kinase 1;IMPDH, inosine 5'-monophosphate dehydrogenase;NDK, nucleoside diphosphate kinase;PAICS, phosphoribosylaminoimidazole carboxylase;PFAS, phosphoribosylformylglycinamidine synthase; PPAT, phosphoribosyl pyrophosphate amidotransferase; PRPS1, phosphoribosyl pyrophosphate synthetase 1. **Figure s3.** Biosynthetic pathway of pyrimidine nucleotides. A graphical depiction of cellular *de novo* biosynthesis of pyrimidines. Blue arrows represent pyrimidine monophosphate synthesis through the salvage pathway. The enzymes that are discussed in the manuscript are colored in red. Abbreviations: CAD, trifunctional CAD enzyme; CMPK, cytidine monophosphate (UMP-CMP) kinase 1;CTPS, CTP synthase; dCK, deoxycytidine kinase; DCTD, deoxycytidine monophosphate deaminase; DHODH, dihydroorotate dehydrogenase; DUT, deoxyuridine triphosphatase; NDK, nucleoside diphosphate kinase;UCK2, uridine-cytidine kinase 2; UMPS, uridine 5'-monophosphate synthase; RNR, ribonucleoside-diphosphate reductase; TK1, thymidine kinase 1; TMPK, thymidylate kinase; TS, thymidylate synthase. **Figure S4.** Cellular folate metabolism. A graphical depiction of cellular pathways utilizing folate cofactos. The enzymes that are discussed in the manuscript are colored in red. Abbreviations: ALDH1L2, aldehyde dehydrogenase 1 family, member L2; ATIC, 5-aminoimidazole-4-carboxamide ribonucleotide formyltransferase;DHF, dihydrofolate; THF, tetrahydrofolate; DHFR, dihydrofolate reductase; DHFRL1, dihydrofolate reductase-like 1; GART, phosphoribosylglycinamide formyltransferase;MTHFD, methylenetetrahydrofolate dehydrogenase; MDHFD1L, methylenetetrahydrofolate dehydrogenase 1-like;MTHFR, methylenetetrahydrofolate reductase; MTHFS, 5,10-methenyltetrahydrofolate synthetase; MTR, 5-methyltetrahydrofolate-homocysteine methyltransferase; SHMT, serine hydroxymethyltransferase; TS, thymidylate synthase.

## Data Availability

All data generated or analyzed during this study are included in this published article.

## Abbreviations

AML: Acute myeloid leukemia
Ara-C: Cytosine arabinoside (cytarabine)
DNR: Daunorubicin
MX: Mitoxantrone
VP-16: Etoposide
ABC: ATP-binding cassette
ENT: Equilibrative nucleoside transporte
CNT: concentrative nucleoside transporter
OCTN1: Organic cation transporter, novel, type 1
dCK: Deoxycytidine kinase
dCMPK: Deoxycytidylate kinase
NDK: Nucleoside diphosphate kinase
CDA: Cytidine deaminase
dCMPD: Deoxycytidine monophosphate deaminase
P-gp: P-glycoprotein
MDR: Multidrug resistance
VEN: Venetoclax
GO: Gemtuzumab ozogamicin
MRP: Multidrug resistance-associated protein
HSCT: Hematopoietic stem cell transplantation
RT PCR: Real-time polymerase chain reaction
GUSB: Glucuronidase β
OS: Overall survival
POLR1D: DNA-directed RNA polymerase I subunit D
P1/2: Patient 1/2
HiDAC: High dose Ara-C
CR: Complete remission
WBC: White blood cells
GR: Good response
LD: Lysosomotropic drug
CLEAR: Coordinated lysosomal expression and regulation
ATP6v1H: V-type proton ATPase subunit H
CTSD: Cathepsin D
MSMO1: Methylsterol monooxygenase 1
HMGCR: 3-hydroxy-3-methylglutaryl-coenzyme A reductase
DNSP: *de novo* synthesis pathway
CAD: Trifunctional CAD enzyme
CTPS1: CTP synthase 1
UMPS: Uridine 5'-monophosphate synthase
TYMS: Thymidylate synthase
AICARFT: Aminoimidazole-4-carboxamide ribonucleotide formyltransferase
RRM1: Ribonucleoside-diphosphate reductase subunit M1
DHFR: Dihydrofolate reductase
GART: Phosphoribosylglycinamide formyltransferase
RFC: Reduced folate carrier
FPGS: Folylpoly-ɣ-glutamate synthetase
AZT: Azidothymidine
HU: Hydroxyurea
MTX: Methotrexate
CAR: Chimeric antigen receptor
