# Supplementary material for: Deciphering molecular mechanisms underlying chemoresistance in relapsed AML patients: towards precision medicine overcoming drug resistance
Source: Cancer Cell Int. 2021 Jan 14;21:53. doi: 10.1186/s12935-021-01746-w (PMC7809753; doi:10.1186/s12935-021-01746-w)
Supplement: Supplementary file 1 — Additional file 1: Figure. S1. Summary of drug metabolism. A graphical depiction of cellular transport, metabolism, and targets of AML chemotherapeutic drugs discussed in the manuscript. Pro-drug intermediates are colored in black, bioactive drugs are colored in light blue, and inactivated metabolites are colored in grey. Abbreviations: AICARFT, aminoimidazole-4-carboxamide ribonucleotide formyltransferase; Ara-C, cytosine arabinoside; ATP6V1H, V-type proton ATPase subunit H; Bcl-2, B-cell leukemia/lymphoma 2; Bcl-XL, apoptosis regulator Bcl-X, long isoform; BCRP, breast cancer resistance protein; CDA, cytidine deaminase; CNT3, concentrative nucleoside transporter 3; CTSD, cathepsin D; dCK, deoxycytidine kinase; dCMPD, deoxycytidylate deaminase; dCMPK, deoxycytidylate kinase; DHFR, dihydrofolate reductase; DNR, daunorubicin;ENT1, esquilibrative nucleoside transporter 1; FPGS, folylpoly-ɣ-glutamate synthetase; GO, Gemtuzumab Ozogamicin; HMGCR, 3-hydroxy-3-methylglutaryl-coenzyme A reductase; Mcl-1, Induced myeloid leukemia cell differentiation protein Mcl-1; MRP, multidrug resistance-associated protein; MSMO1, methylsterol monooxygenase 1;MTX-polyG, methotrexate polyglutamate; MX, mitoxantrone; NDK, nucleoside diphosphate kinase; OCTN1, organic cation transporter, novel, type 1; PCFT, proton coupled folate transporter; P-gp, P-glycoprotein; PN-I, cytosolic 5-nuleotidase 3A; RFC, reduced folate carrier; TS, thymidylate synthase; VEN, venetoclax; VP-16, etoposide. Figure S2. Biosynthetic pathway of purine nucleotides. A graphical depiction of cellular de novo biosynthesis of purines. The enzymes that are discussed in the manuscript are colored in red. Abbreviations: ADSL, adenylosuccinate lyase;ADSS, adenylosuccinate synthase; AK, adenylate kinase; ATIC, 5-aminoimidazole-4-carboxamide ribonucleotide formyltransferase; GART, phosphoribosylglycinamide formyltransferase;GMPS, guanine monphosphate synthase;GUK1, guanylate kinase 1;IMPDH, inosine 5'-monophosphate dehydrogen [file 12935_2021_1746_MOESM1_ESM.pdf]

Deciphering molecular mechanisms underlying chemoresistance in relapsed AML patients:

Towards precision medicine overcoming drug resistance

Additional file 1

Graphical description of drug metabolism, nucleotide biosynthesis and folate metabolism pathways.

Additional Figure S1: Summary of drug metabolism (detailed in Table 1 in the main text)……..…2

Additional Figure S2: Biosynthetic pathway of purines………………………………………….…3

Additional Figure S3: Biosynthetic pathway of pyrimidines……………………………………...…4

Additional Figure S4: Folate metabolism pathways……………………………………………........5

Summary of drug metabolism

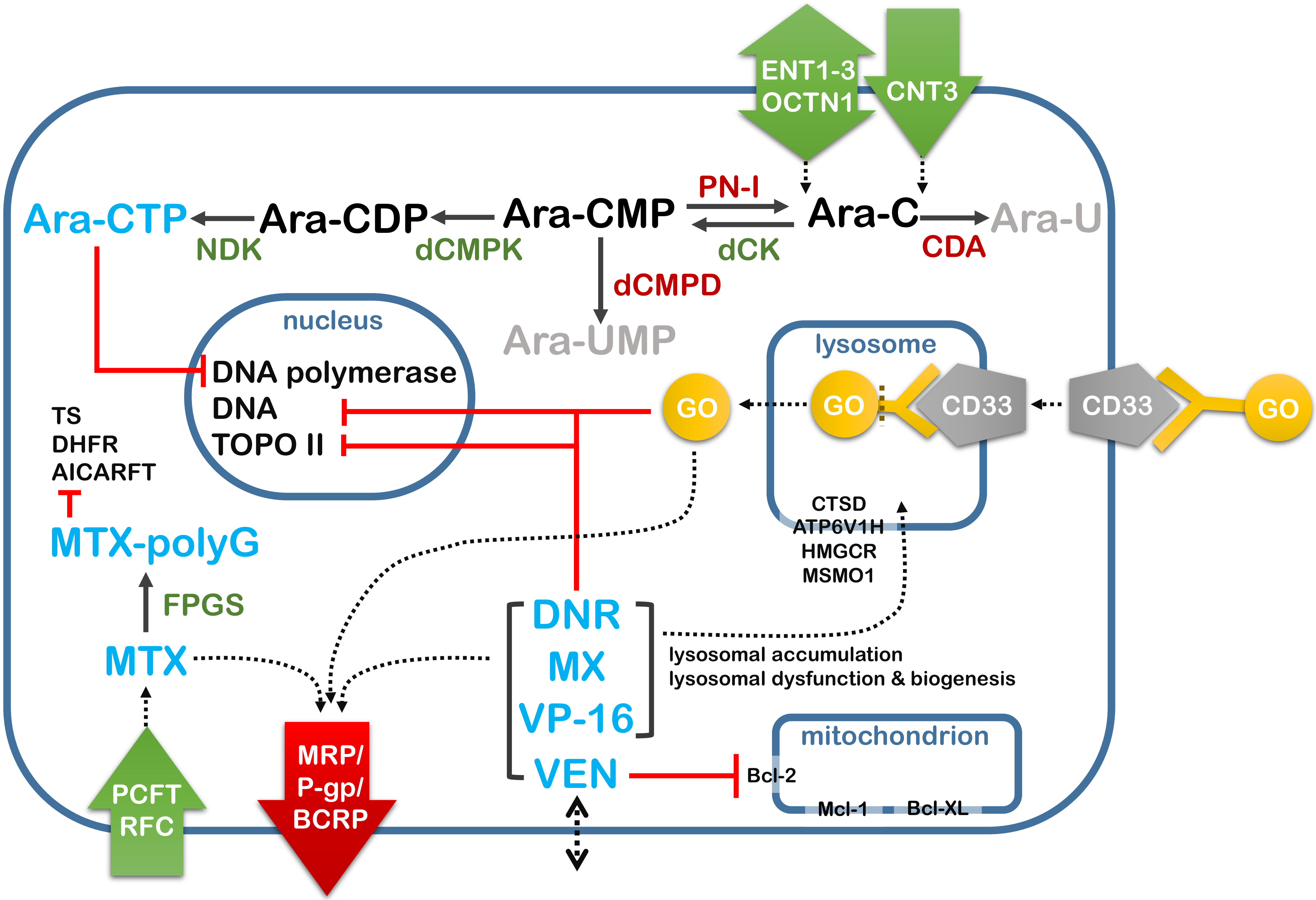

Additional Figure S1: Summary of drug metabolism. A graphical depiction of cellular transport, metabolism, and targets of AML chemotherapeutic drugs discussed in the manuscript. Pro-drug intermediates are colored in black, bioactive drugs are colored in light blue, and inactivated metabolites are colored in grey. Abbreviations: AICARFT, aminoimidazole-4-carboxamide ribonucleotide formyltransferase; Ara-C, cytosine arabinoside; ATP6V1H, V-type proton ATPase subunit H; Bcl-2, B-cell leukemia/lymphoma 2; Bcl-XL, apoptosis regulator Bcl-X, long isoform; BCRP, breast cancer resistance protein; CDA, cytidine deaminase; CNT3, concentrative nucleoside transporter 3; CTSD, cathepsin D; dCK, deoxycytidine kinase; dCMPD, deoxycytidylate deaminase; dCMPK, deoxycytidylate kinase; DHFR, dihydrofolate reductase; DNR, daunorubicin; ENT1, equilibrative nucleoside transporter 1; FPGS, folylpoly-ɣ-glutamate synthetase; GO, Gemtuzumab Ozogamicin; HMGCR, 3-hydroxy-3-methylglutaryl-coenzyme A reductase; Mcl-1, Induced myeloid leukemia cell differentiation protein Mcl-1; MRP, multidrug resistance-associated protein; MSMO1, methylsterol monooxygenase 1; MTX-polyG, methotrexate polyglutamate; MX, mitoxantrone; NDK, nucleoside diphosphate kinase; OCTN1, organic cation transporter, novel, type 1; PCFT, proton coupled folate transporter; P-gp, P-glycoprotein; PN-I, cytosolic 5-nuleotidase 3A; RFC, reduced folate carrier; TS, thymidylate synthase; VEN, venetoclax; VP-16, etoposide.

Purine biosynthesis

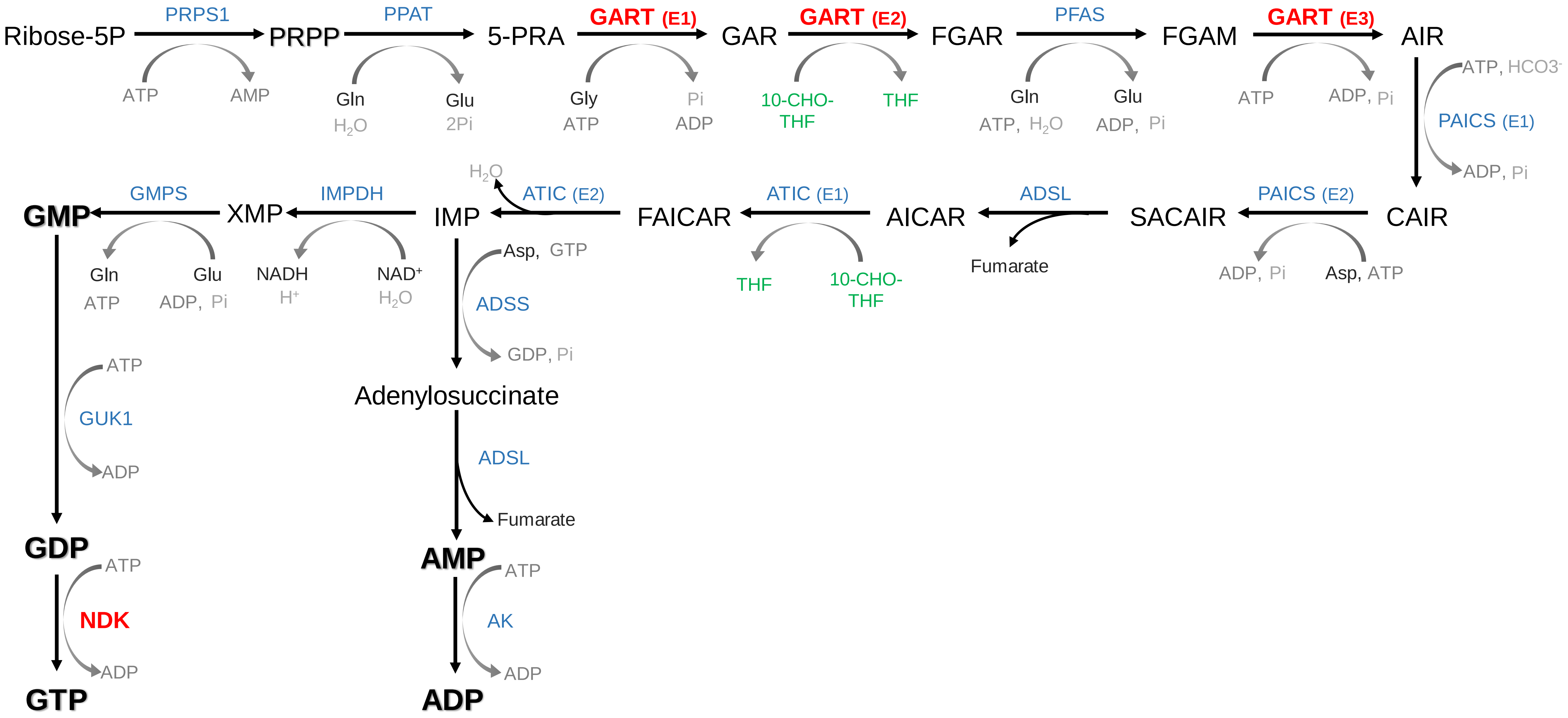

Additional Figure S2: Biosynthetic pathway of purine nucleotides. A graphical depiction of cellular *de novo* biosynthesis of purines. The enzymes that are discussed in the manuscript are colored in red. Abbreviations: ADSL, adenylosuccinate lyase; ADSS, adenylosuccinate synthase; AK, adenylate kinase; ATIC, 5-aminoimidazole-4-carboxamide ribonucleotide formyltransferase; GART, phosphoribosylglycinamide formyltransferase; GMPS, guanine monphosphate synthase; GUK1, guanylate kinase 1; IMPDH, inosine 5'-monophosphate dehydrogenase; NDK, nucleoside diphosphate kinase; PAICS, phosphoribosylaminoimidazole carboxylase; PFAS, phosphoribosylformylglycinamidine synthase; PPAT, phosphoribosyl pyrophosphate amidotransferase; PRPS1, phosphoribosyl pyrophosphate synthetase 1.

Pyrimidine biosynthesis

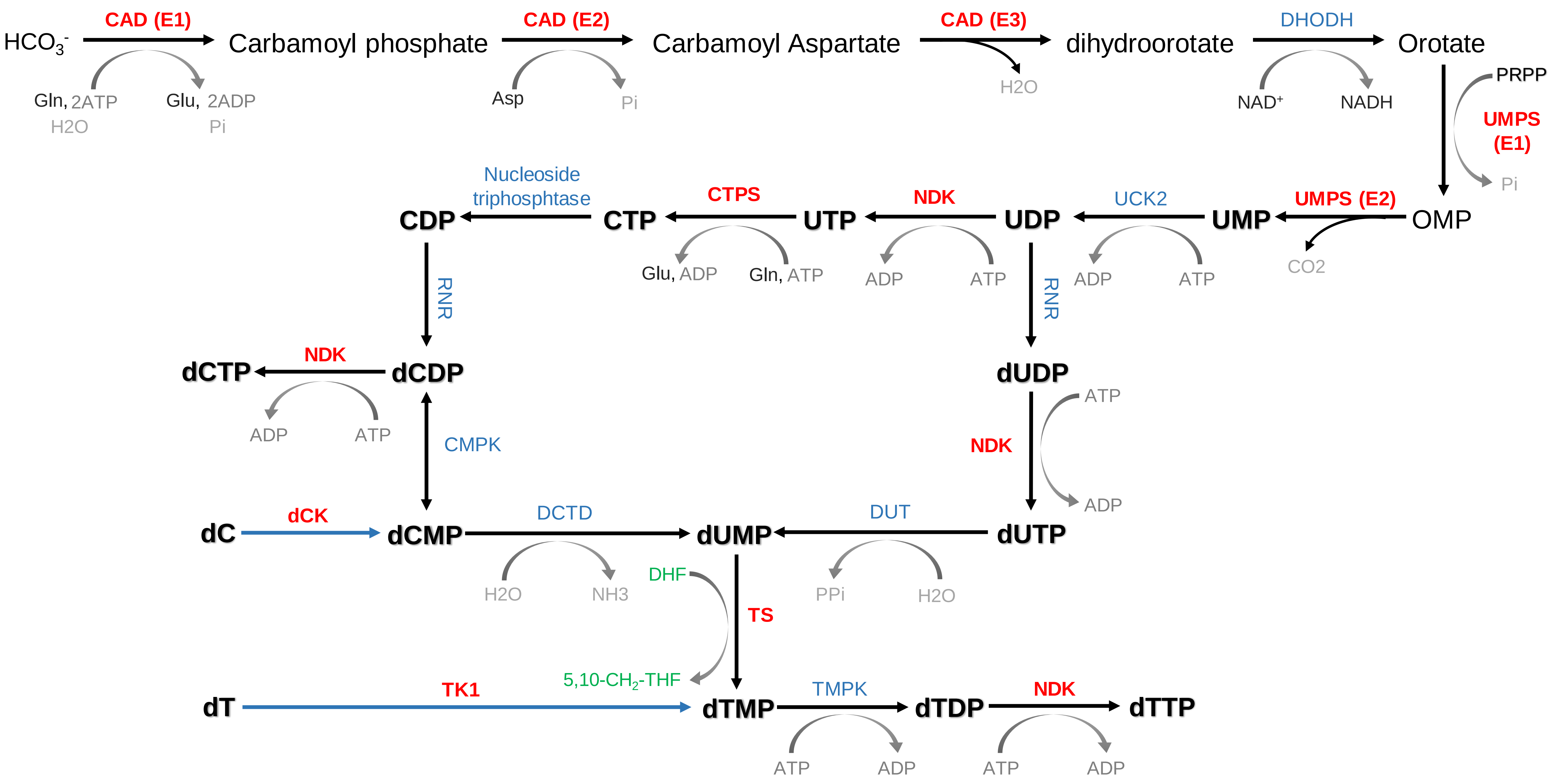

Additional Figure S3: Biosynthetic pathway of pyrimidine nucleotides. A graphical depiction of cellular *de novo* biosynthesis of pyrimidines. Blue arrows represent pyrimidine monophosphate synthesis through the salvage pathway. The enzymes that are discussed in the manuscript are colored in red. Abbreviations: CAD, trifunctional CAD enzyme; CMPK, cytidine monophosphate (UMP-CMP) kinase 1; CTPS, CTP synthase; dCK, deoxycytidine kinase; DCTD, deoxycytidine monophosphate deaminase; DHODH, dihydroorotate dehydrogenase; DUT, deoxyuridine triphosphatase; NDK, nucleoside diphosphate kinase; UCK2, uridine-cytidine kinase 2; UMPS, uridine 5'-monophosphate synthase; RNR, ribonucleoside-diphosphate reductase; TK1, thymidine kinase 1; TMPK, thymidylate kinase; TS, thymidylate synthase.

Folate Metabolism

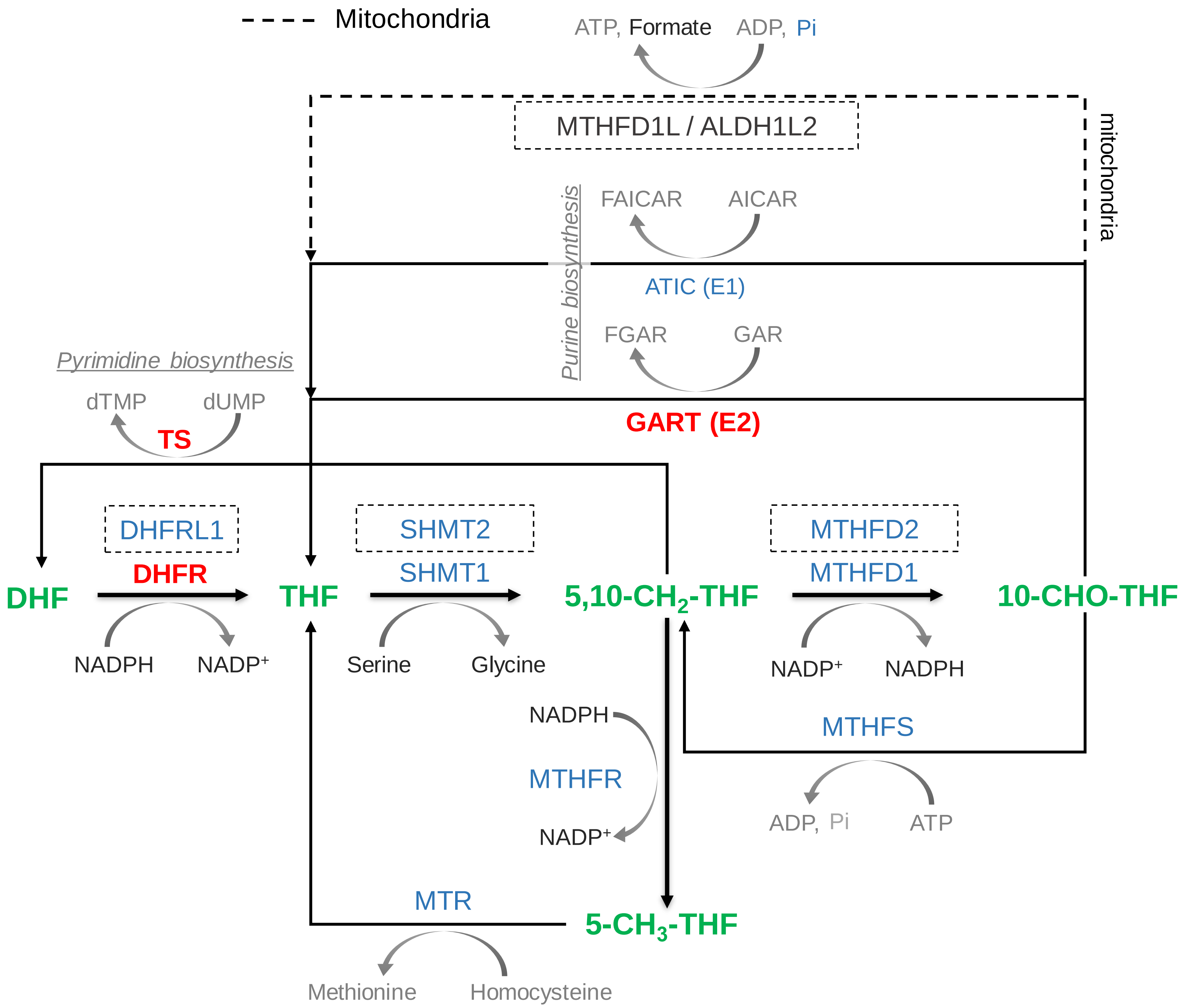

Additional Figure S4: Cellular folate metabolism. A graphical depiction of cellular pathways utilizing folate cofactos. The enzymes that are discussed in the manuscript are colored in red. Abbreviations: ALDH1L2, aldehyde dehydrogenase 1 family, member L2; ATIC, 5-aminoimidazole-4-carboxamide ribonucleotide formyltransferase; DHF, dihydrofolate; THF, tetrahydrofolate; DHFR, dihydrofolate reductase; DHFRL1, dihydrofolate reductase-like 1; GART, phosphoribosylglycinamide formyltransferase; MTHFD, methylenetetrahydrofolate dehydrogenase; MDHFD1L, methylenetetrahydrofolate dehydrogenase 1-like; MTHFR, methylenetetrahydrofolate reductase; MTHFS, 5,10-methenyltetrahydrofolate synthetase; MTR, 5-methyltetrahydrofolate-homocysteine methyltransferase; SHMT, serine hydroxymethyltransferase; TS, thymidylate synthase.
